# Microfluidic models of the neurovascular unit: a translational view

**DOI:** 10.1186/s12987-023-00490-9

**Published:** 2023-11-27

**Authors:** Nienke R. Wevers, Helga E. De Vries

**Affiliations:** 1 MIMETAS BV, De Limes 7, Oegstgeest, 2342 DH The Netherlands; 2 Amsterdam UMC location Vrije Universiteit Amsterdam, Amsterdam Neuroscience – Neuroinfection and Neuroinflammation, De Boelelaan 1117, Amsterdam, the Netherlands

**Keywords:** Blood-brain barrier, Neurovascular unit, Microfluidics, Organ-on-a-chip, In vitro models

## Abstract

The vasculature of the brain consists of specialized endothelial cells that form a blood-brain barrier (BBB). This barrier, in conjunction with supporting cell types, forms the neurovascular unit (NVU). The NVU restricts the passage of certain substances from the bloodstream while selectively permitting essential nutrients and molecules to enter the brain. This protective role is crucial for optimal brain function, but presents a significant obstacle in treating neurological conditions, necessitating chemical modifications or advanced drug delivery methods for most drugs to cross the NVU. A deeper understanding of NVU in health and disease will aid in the identification of new therapeutic targets and drug delivery strategies for improved treatment of neurological disorders.

To achieve this goal, we need models that reflect the human BBB and NVU in health and disease. Although animal models of the brain’s vasculature have proven valuable, they are often of limited translational relevance due to interspecies differences or inability to faithfully mimic human disease conditions. For this reason, human in vitro models are essential to improve our understanding of the brain’s vasculature under healthy and diseased conditions. This review delves into the advancements in in vitro modeling of the BBB and NVU, with a particular focus on microfluidic models. After providing a historical overview of the field, we shift our focus to recent developments, offering insights into the latest achievements and their associated constraints. We briefly examine the importance of chip materials and methods to facilitate fluid flow, emphasizing their critical roles in achieving the necessary throughput for the integration of microfluidic models into routine experimentation. Subsequently, we highlight the recent strides made in enhancing the biological complexity of microfluidic NVU models and propose recommendations for elevating the biological relevance of future iterations.

Importantly, the NVU is an intricate structure and it is improbable that any model will fully encompass all its aspects. Fit-for-purpose models offer a valuable compromise between physiological relevance and ease-of-use and hold the future of NVU modeling: as simple as possible, as complex as needed.

## Introduction

### The brain and neurological diseases

**Fig. 1:**
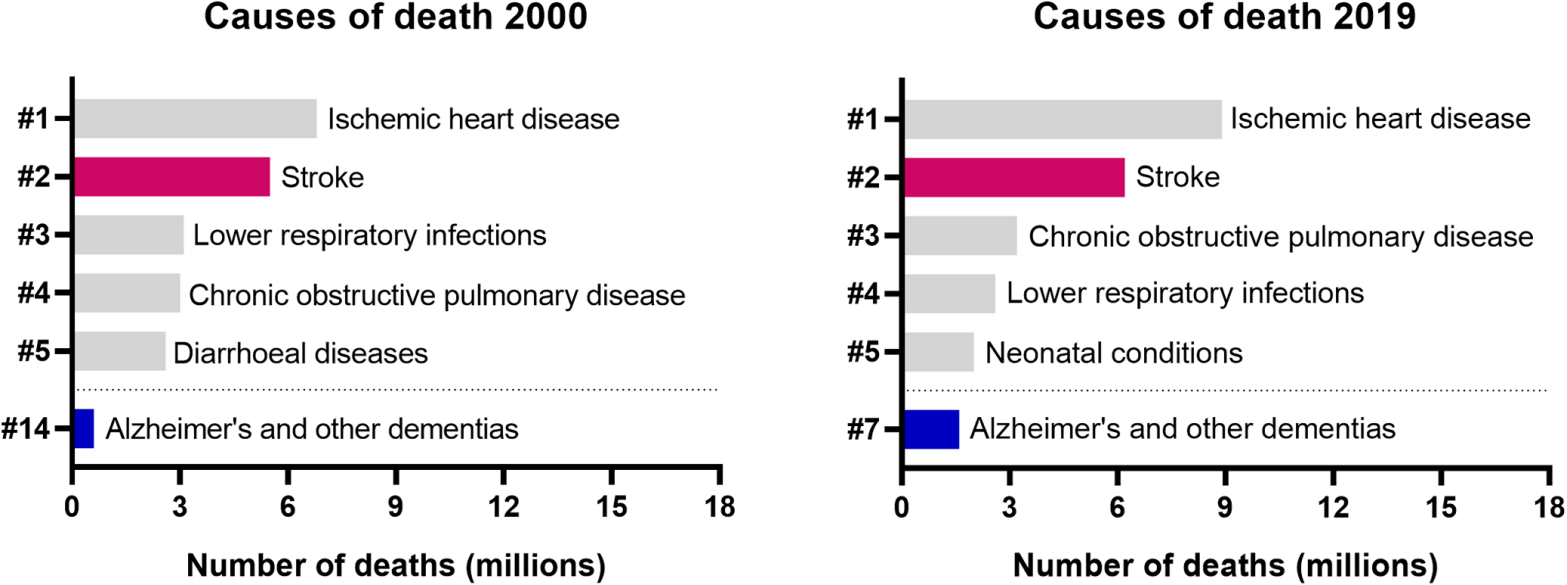
Global causes of death according to the World Health Organization’s Global Health Estimates. Data includes all sexes and age groups. Importantly, numbers strongly differ between different continents (not shown here). While stroke is among the most common causes of death in all continents, Alzheimer’s disease and other dementias are less prevalent in developing regions in which the average life expectancy is lower

The central nervous system (CNS) is essential for proper body functioning and our cognitive performance. For this reason, impaired CNS function can lead to a myriad of diseases and symptoms. According to data from the World Health Organization, neurological and psychiatric disorders are in the top three of life-threatening diseases. For instance, stroke is the second cause of death worldwide and a major cause of adult disability [1, 2]. Stroke is also considered a significant risk factor for developing dementia, among several other factors [3, 4]. The number of deaths caused by Alzheimer’s disease and other dementias more than doubled between 2000 and 2019, making it the 7th leading cause of death globally (Fig. 1).

Despite decades of research into CNS disorders, we still do not fully understand the underlying disease mechanisms. This lack of understanding is in part due to the tremendous complexity of the brain and its vasculature. Current estimates state that the brain contains over 80 billion neurons [5], a myriad of glial cells, and over 600 km of vasculature [6]. Furthermore, the brain and its vasculature are highly heterogeneous. Different areas of the brain present with distinct microenvironments that are adapted to the local needs and thus exert different functions [7–10]. Improved understanding of the brain and its vasculature will aid the discovery of new treatments that improve patients’ quality of life or even cure diseases.

### The neurovascular unit

The brain needs a homeostatic environment to function properly. This protective environment is ensured by specialized endothelial cells that make up the vasculature of the brain, forming a tight blood-brain barrier (BBB). The BBB prevents large, polar substances and potentially neurotoxic compounds from the circulation from passively diffusing into the brain. Essential nutrients that cannot pass the BBB via diffusion, such as glucose, enter the brain via specialized influx transporters [11, 12]. Harmful molecules, on the other hand, are cleared from the brain via efflux transporters [13, 14].

**Fig. 2:**
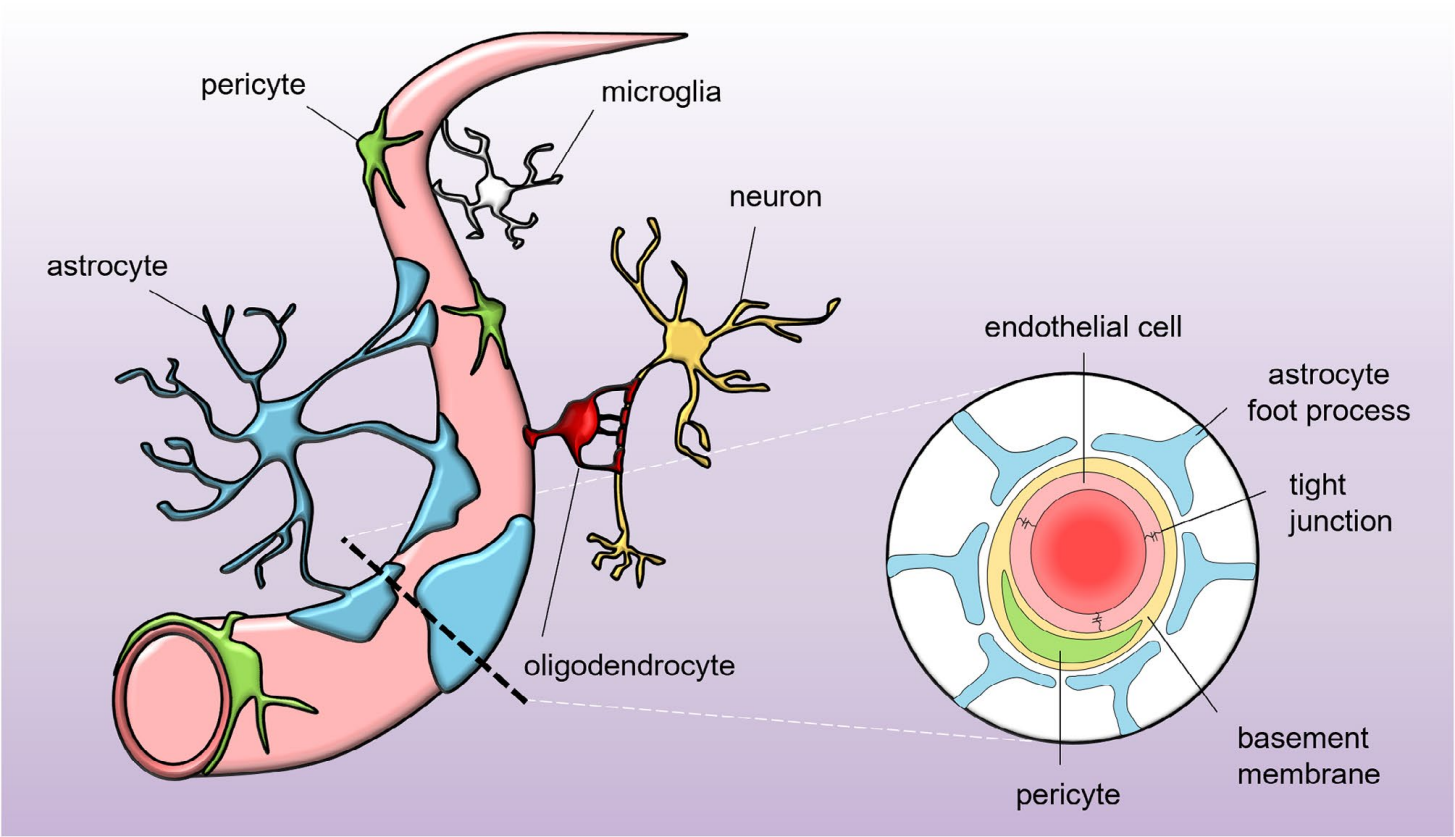
Schematic representation of the neurovascular unit. The vasculature of the brain consists of specialized brain endothelial cells that form a blood-brain barrier. The endothelial cells are embedded in the basement membrane, a non-cellular component consisting of extracellular matrix proteins, together with pericytes, which make direct contact with the endothelial cells. Astrocytes connect to the structure made up of basement membrane, pericytes, and endothelial cells via their foot processes. In addition, astrocytes have extensive contact with neurons. Oligodendrocytes provide myelination to neuronal axons, which is essential for proper transduction of neuronal signals. Microglia are the resident immune cells of the CNS

The endothelial cells of the BBB are sealed by proteins spanning the clefts between adjacent cells, forming tight junctions (TJs) and adherens junctions (AJs) [15, 16]. The functioning of TJs and AJs is supported by other cell types, which are in direct contact with the brain endothelial cells, such as pericytes and astrocytes [17, 18]. These supporting cell types are essential for maintaining barrier function and transport across the BBB. The entire system contributing to BBB function is referred to as the neurovascular unit (NVU), and includes brain endothelial cells, pericytes, and astrocytes, but also neurons, oligodendrocytes, microglia, and the basement membrane [12, 19] (Fig. 2).

Improving our knowledge of BBB and NVU functioning is of utmost importance for two reasons. First, BBB dysfunction is a common feature across almost all CNS disorders [20–22]. Impaired barrier function is often accompanied by endothelial inflammation, thereby facilitating infiltration of circulating immune cells into the CNS [23–25]. The immune cells release inflammatory mediators, such as cytokines, free radicals, and matrix metalloproteinases, which further worsen the barrier function and disease state [26–29]. A better understanding of the processes involved in healthy BBB functioning and how these are disturbed in brain diseases will help us find new targets for treatment. Second, while the BBB protects the brain from harmful substances in the circulation, it also poses a major challenge when it comes to treating brain diseases [30, 31]. As the BBB only allows small, lipid soluble molecules to pass freely, most drugs require advanced drug delivery strategies to enter the brain [31–33]. A better understanding of BBB and NVU functioning will shed light on new techniques and drug delivery strategies to effectively target drugs into the brain to treat CNS disorders. To achieve this goal of improved understanding of NVU functioning in health and disease and advance our knowledge of drug targeting to the brain, we need models that reflect the human NVU in health and disease.

## Modeling the neurovascular unit

### The first in vitro NVU models

While animal models have proven useful in studying the brain’s vasculature, the use of animals is costly, time-consuming, and ethically undesirable. Furthermore, data obtained from animal studies often results in poor translatability to the human physiology due to interspecies differences [34–36]. While the cellular composition of the NVU is similar between humans and rodents, other important features are not. The expression level of many relevant junctional proteins and transporters differs between species, which results in differences in drug uptake and efflux. Additionally, drug distribution across the brain may differ due to differences in lipid composition of the brain between species. Importantly, animal models of disease often fail to account for alterations in NVU function related to aging or neurological disease and have reported conflicting results [35–37]. While in vitro models of the NVU do not display the level of complexity as animal models do, they do allow for the use of human cells, in highly controlled settings, at lower cost, and within shorter time frames.

The first attempt at in vitro NVU modeling started with the isolation of brain capillaries from rats [38]. Since then, many studies of primary rodent, porcine, bovine, and later human brain endothelial cells have been reported, using both monocultures and co-cultures with supporting cell types [39–44]. Later, immortalized cell lines of human brain endothelial cells were established [45, 46], followed by protocols for stem-cell derived models [47, 48] and self-assembling spheroids [49–51].

As cellular models of the NVU progressed [52], so did cell culture platforms [37, 53]. Initially, studies were performed using traditional two-dimensional (2D) culture systems [54]. Aiming to improve physiological relevance and complexity, the first models using a Transwell system were developed [42, 55]. In this system, brain endothelial cells are cultured on one side of a semi-permeable membrane and supporting cells such as astrocytes or pericytes on the other. Although the Transwell systems presented a step forward in physiological NVU modeling, the lack of flow and direct cell-cell contact, and the presence of a membrane posed limitations. In response to those unmet needs, microfluidic platforms made their appearance in the field of NVU modeling [37].

### Debut of microfluidic models

Microfluidic platforms make use of tissue culture chips comprising small channels that allow the development of layered three-dimensional (3D) cell cultures under flow [56]. The first microfluidic NVU models consisted of hollow fiber apparatuses to culture bovine aortic endothelial cells and rat glioma cells under shear stress [57–59]. These models confirmed previous reports of beneficial effects of co-culture and for the first time reported that culture under flow improves barrier properties of NVU models.

Following the hollow fiber apparatuses, microfluidic polydimethylsiloxane (PDMS) based chips using planar structures were employed. Booth and colleagues developed the first NVU model in such a chip, using murine endothelial cells and astrocytes, establishing a much thinner membrane than previously used in the hollow fiber apparatuses (10 μm versus 150 μm, respectively) [60]. The thinner membranes allowed for closer cell-cell contact in co-culture setups, and similar approaches were taken in many subsequent studies using primary cells and cell lines from various species [61–68].

The most recent microfluidic NVU models still show resemblance to the chip reported by Booth et al., but nowadays special focus is on all-human models, using primary material [69], or iPSC-derived cells [70, 71], allowing for potential use in personalized therapies.

## Increased throughput for routine experimentation

While many microfluidic platforms have been developed for complex NVU modeling, most of these are very low in throughput and cumbersome to use. There is a need for higher throughput, more user-friendly platforms that could unite microfluidic NVU models with routine experimentation, evaluation of compound toxicity, and study of drug candidates’ ability to enter the brain [72–74]. Among other factors, chip materials and approaches to accommodate fluid flow through microfluidic chips are important considerations in achieving the necessary throughput.

### Chip materials

Polydimethylsiloxane (PDMS) played a pivotal role in the foundational research within the organ-on-a-chip field and continues to be a predominant material, with the majority of organ-on-a-chip devices still relying on PDMS as their primary structural and cell-interacting component [75–77]. The material’s transparency allows for visualization of cells’ growth and behavior within microfluidic channels. Additionally, PDMS is biocompatible, economical, and exhibits high elasticity, which enables the fabrication of microfluidic devices with complex geometries and tight sealing between different channels. However, despite its numerous advantages, PDMS also poses several challenges when used in cell culture applications [76, 78, 79]. PDMS is auto-fluorescent, which may complicate fluorescence-based assays. Moreover, PDMS is incompatible with organic solvents and is intrinsically hydrophobic. The hydrophobic properties hinder cell adhesion, introducing challenges in tissue engineering. Additionally, these properties result in the uptake of hydrophobic molecules, including cell culture media components, signaling compounds, and therapeutics, which can impact the reproducibility and accuracy of experimental results [80–84]. Lastly, the production process of PDMS chips itself as well as methods to mitigate its hydrophobicity are generally difficult to incorporate in large-scale production [76, 78, 79]. For this reason, PDMS-based chips are usually low in throughput [85].

Many microfluidic platforms combine different materials to improve the chips’ properties, such as compatibility with microscopic imaging, biocompatibility, chemical compatibility, and hydrophobicity. Three materials commonly used in microfluidic chips alongside PDMS are silica nitride (SiN), polyethylene terephthalate (PET), and polycarbonate (PC). While these materials can be used as standalone materials for chip fabrication, they are more commonly used in combination with PDMS chips, often as a membrane to partition a chip’s microfluidic channels. An advantage of SiN is its transparency across a wide range of wavelengths, making it ideal for imaging and fluorescence-based assays. This advantage was leveraged in a recent publication of an in vitro BBB model to investigate intracellular trafficking of antibodies using high resolution imaging [86]. An advantage of PET is its low cost and great chemical stability, allowing exposure to many solvents and reagents. Chips incorporating PET membranes were used to model the NVU in recent work by Walter et al. [67] and Park et al. [71]. Unlike PDMS and PET, PC is hydrophilic, allowing easier cell adhesion to its surface and promoting fluid flow. Furthermore, it is known for its robust mechanical properties, making it a durable choice for creating membrane and microfluidic structures. Achyuta and colleagues employed a PDMS chip containing a PC membrane to establish a rat NVU model [63].

**Fig. 3:**
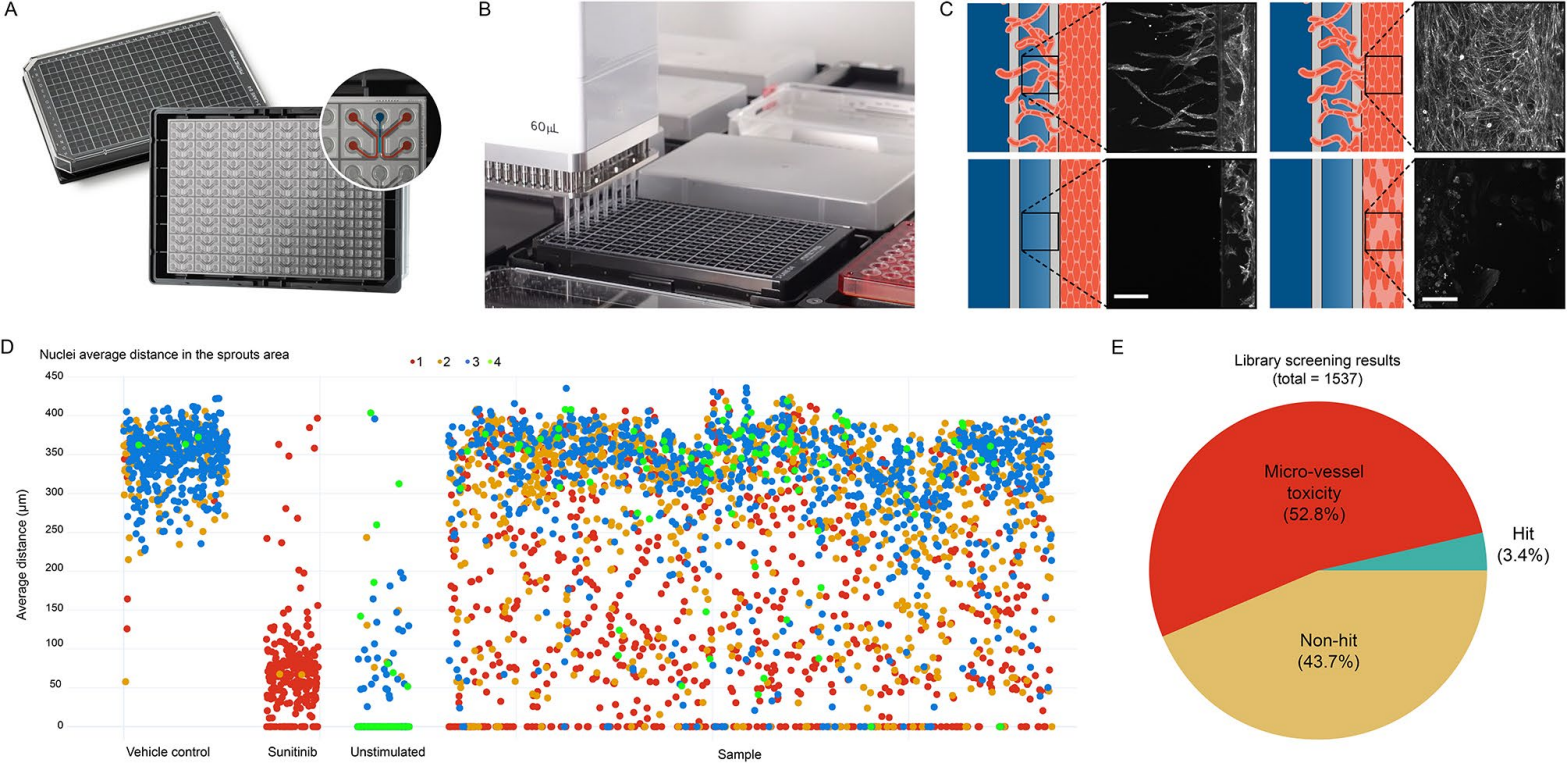
Screen of 1537 compounds using a microfluidic cell culture platform. **(a)** The OrganoPlate 3-lane harbors 64 chips which each allow culture of a miniaturized tissue. **(b)** The platform is compatible with standard lab equipment and automation allowing for sufficient throughput for compound screening. **(c)** A tubule of HUVECs (red) was grown in each chip against an extracellular matrix gel, after which an angiogenic cocktail was added on the opposite side of the gel to create a gradient (dark blue) of angiogenic factors. In response to this gradient, angiogenic sprouts form in the gel region. Inhibition of angiogenesis was assessed in presence of 1537 kinase inhibitors and positive control sunitinib. For each compound, the anti-angiogenic effect (panel I) and toxicity in the parental HUVEC micro-vessel (panel II) was evaluated. **(d)** Average distance of the ten furthest nuclei (representing angiogenic sprouts) with respect to the parental HUVEC micro-vessel in µm. Each dot represents a chip and was color coded for toxicity as assessed by micro-vessel actin network integrity with score of 1 (fully degraded HUVEC tubule) to 4 (fully intact HUVEC tubule). **(e)** Pie chart showing percentage of hits, non-hits and compounds that showed micro-vessel toxicity. Figure was adapted from Soragni et al. (2023) and used in compliance with the requirements of the Creative Commons CC-BY license under which it was published

There are many other materials that can be used for the fabrication of organs-on-chips beyond those discussed above. Comprehensive overviews of different chip designs, materials, and approaches to microfluidic NVU modeling were recently provided in various reports [37, 87–89]. One disadvantage that most chips, comprised of different materials, have in common is their low throughput, which prevents their adoption in routine experimentation and compound screening [85, 90]. In response to this unmet need, efforts have been made to enable microfluidic cell culture at higher throughputs. Trietsch at al. presented a microfluidic tissue culture platform comprised of glass and polystyrene that allows parallel culture of 40 organs-on-chips in a 384-well plate format [91]. The microfluidic system, called the OrganoPlate, is compatible with automation and standard laboratory and imaging equipment. Soragni and colleagues recently performed a screen of 1537 compounds on human umbilical vein endothelial cells (HUVECs) cultured in this microfluidic system and assessed toxicity and efficacy in inhibiting the formation of angiogenic sprouts [92]. This work led to the identification of ~ 50 safe and efficacious hits and shows the potential of microfluidic models in routine experimentation and even compound screening (Fig. 3).

### Fluid flow

While the brain only accounts for approximately 2% of the total body mass, it demands ~ 20% of the body’s oxygen and ~ 25% of the body’s glucose consumption. Oxygen and nutrients are supplied by cerebral blood flow, which accounts for ~ 15% of the cardiac output, equaling approximately 750 milliliters per minute in rest state. Cerebral blood flow is dynamic and is elevated in case of increased neuronal activity in a specific region of the brain [93, 94].

Traditional models of the NVU do not incorporate fluid flow and culture brain endothelial cells under static conditions. Although some have reported that unlike other endothelial cells, brain endothelial cells do not align under flow [95, 96], the general view is that perfused culture better mimics the environment found in vivo and improves barrier function [97–99]. Siddharthan and colleagues compared barrier function of primary human brain endothelial cells (BECs) cultured static or under flow using a hollow fiber-apparatus. In response to shear stress in the flow apparatus, BECs showed reduced BBB permeability [98]. Similar findings were presented by Cucullo and colleagues, who observed upregulation of junctional proteins in primary human BECs cultured under flow compared to static culture. The BECs cultured under flow showed reduced BBB permeability, decreased cell division, and increased expression of drug and nutrient transporters [99]. Following hollow-fiber apparatuses, planar chips were introduced to the field of NVU modeling, and allowed for closer contact between endothelial cells and supporting cells [60]. Following the initial work by Booth and colleagues, many others have confirmed positive effects of flow on in vitro cultured brain endothelial cells, employing primary material [66, 67], cell lines [61, 62, 67], and stem cell derived cells [70].

Using a high-throughput microfluidic cell culture platform, we have previously shown improved cell viability and barrier formation in response to perfused culture. An immortalized brain endothelial cell line showed improved junctional organization and decreased permeability when cultured under bidirectional, gravity-driven flow compared to static conditions [100]. This improvement was likely caused by a continuous supply of oxygen and nutrients rather than by shear stress. Further improved barrier function was obtained in the same microfluidic platform in a later publication, in which a vessel of primary human brain microvascular endothelial cells was shown to be tight for small molecule sodium fluorescein (0.45 nm) [101]. The shear stress used in these models (~ 1.2 dyne/cm^2^) is low compared to the shear stress experienced by vessels of similar diameter (~ 300 μm) in vivo [102, 103], but within the range reported for post-capillary venules (1–6 dyne/cm^2^, 20–50 μm diameter) [99, 103, 104]. Furthermore, the flow in these models was bidirectional, while flow in vivo is of unidirectional nature and flow disturbances are associated with diminished vascular health [105]. Increased shear stress and unidirectional flow have been reported for other microfluidic systems by employing fluid flow induced by pumps and syringes [71, 96, 106]. While the resulting fluid flow is more physiologically relevant, the use of pump-based flow comes at the cost of strongly reduced ease of use and throughput. For this reason, physiological relevance and practical considerations must be weighed for each specific research question when selecting a platform for in vitro modeling.

## Increased biological complexity

### Incorporation of microglia

In vitro NVU models initially focused primarily on capturing the endothelial component of the BBB in the form of brain endothelial cells. Over time, supporting cell types such as astrocytes, pericytes, and neurons were added to these models. More recently, increased emphasis is placed on including the brain’s resident immune cells, which are called microglia. Microglia are derived from progenitor cells in the yolk sac, and account for approximately 10% of all cells in the CNS [107, 108]. Upon brain injury or immunological stimuli, resting microglia (M0) undergo several changes and become activated. Early studies hypothesized that following CNS injury, microglia initially shift to a deleterious pro-inflammatory state (M1), followed by a shift to a protective anti-inflammatory state (M2) [109, 110]. More recent work, however, has shown that this view is too simplistic and that microglia activation is a highly complex and dynamic process, with microglia being able to switch from a pro-inflammatory to an anti-inflammatory state and vice versa [111, 112].

Studies have reported substantial interplay between the NVU’s endothelial cells and microglia, both in health and disease. During development, microglia mediate cerebral angiogenesis and stabilization of newly formed blood vessels [113]. After development, resting microglia are found in close proximity to the brain vasculature and monitor blood-brain barrier integrity and entrance of solutes from the circulation into the brain [22]. Recent studies also suggest that microglia contribute to BBB maintenance directly by expression of tight junction protein claudin-5 [114].

Extensive research has been done into the interplay between brain endothelial cells and microglia in disease state. Following BBB disruption, for example due to ischemic stroke, microglia become activated. A recent review by Thurgur & Pinteaux noted four mechanisms for microglia activation following BBB disruption: (1) via factors expressed by endothelial cells, (2) via extravasation of circulating immune cells into the brain, (3) via factors derived from pericytes and remodeling of extracellular matrix proteins, and (4) via microglial priming in long-term inflammation [115].

Conversely, activation of microglia has been shown to affect BBB permeability and functioning. Jolivel and colleagues reported increased association of microglia with cerebral blood vessels in a mouse model of ischemic stroke, followed by local activation of endothelium, phagocytosis of endothelial cells, and BBB breakdown [116]. In line with these findings, Sumi and colleagues showed increased BBB permeability following activation of microglia in an in vitro rat model [117]. The authors suggested production of reactive oxygen species by activated microglia and subsequent disruption of tight junctions as an underlying mechanism. Other studies have implicated microglia-released interleukin 1 beta (IL-1β) in down-regulation of BBB tight junction proteins [118, 119]. A similar relationship is described for microglia-released tumor necrosis factor alpha (TNFα) [120, 121]. In addition to reactive oxygen species, cytokines, and chemokines, activated microglia also produce matrix metalloproteinases (MMPs), which contribute to disruption of BBB basement membrane and tight junctions [122, 123].

BBB breakdown and microglial activation show intensive interplay and are key hallmarks of many neurological diseases, including stroke, Alzheimer’s disease, Parkinson’s disease, and multiple sclerosis (MS) [20–22]. Inclusion of microglia in in vitro models of the NVU can help further elucidate key mechanisms in neurological disease etiology and aid in finding new therapeutic targets. Lyu and colleagues employed a human microglia cell line to study stroke in a human NVU-on-a-chip and found that both pro- and anti-inflammatory markers were induced following ischemic stroke, as observed in in vivo studies [124]. The same cell line was used by Pediaditakis et al. in a microfluidic NVU model alongside brain endothelial-like cells, astrocytes, and neurons. This study reported a decrease in permeability of brain endothelial-like cells as well as increased cytokine production in response to a TNFα trigger in presence of microglia [125]. In recent years, focus has been on obtaining microglia from induced pluripotent stem cells (iPSCs) [126], aiming to increase biological relevance and enable patient-derived models. To the best of our knowledge, no reports have emerged regarding the incorporation of human iPSC-derived microglia into microfluidic NVU models as of yet.

### The role of circulating immune cells

**Fig. 4:**
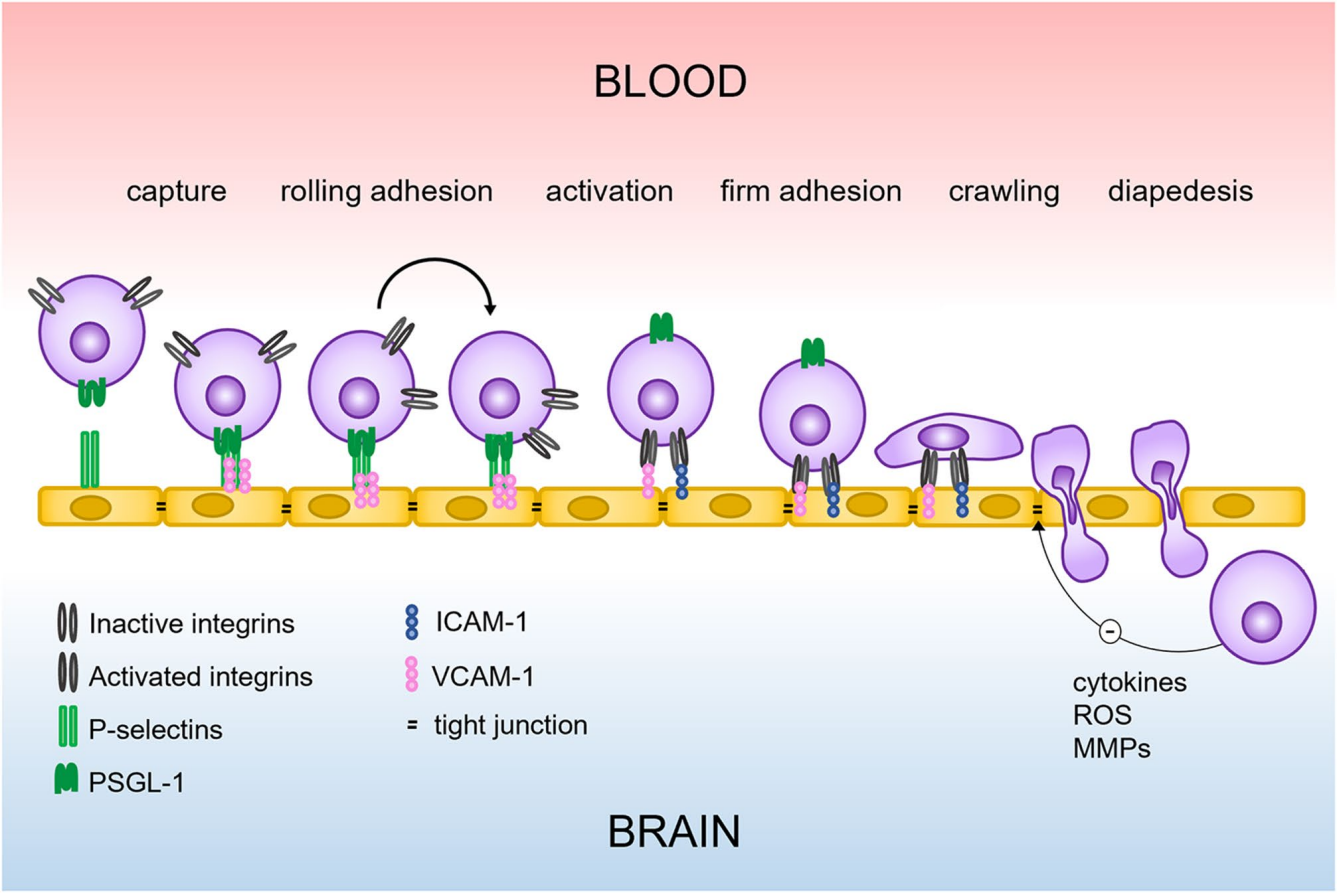
Entry of circulating immune cells into the brain. Entry of immune cells from the periphery into the CNS occurs through a multi-step process, which is initiated by leukocyte capture by the endothelium. Communication between chemokines and chemokine receptors causes activation of leukocytic integrins and enables their interaction with endothelial ICAM-1 and VCAM-1, creating a firm adhesion. After this adhesion is established, the leukocyte crawls along the endothelium – again mediated by chemokine expression – until it enters the brain either through an endothelial cell (transcellular diapedesis) or via an inter-endothelial junction (paracellular diapedesis). After entering the brain, the leukocyte can secrete various molecules – such as cytokines, free radicals, and matrix metalloproteinases – that weaken the tight junctions, change the BBB’s transport properties or degrade the basement membrane, thus further reducing the integrity of the blood-brain barrier. Figure was originally published by Wevers & De Vries (2015) and reused for this manuscript with the copyright holder’s permission

Although circulating immune cells are not part of the NVU, their role in neurological disease and NVU function cannot be ignored. As previously mentioned, BBB disruption is observed in most neurological diseases [12, 20, 127]. Disruption of the barrier coincides with entrance of immune cells from the systemic circulation into the brain [24, 25] (Fig. 4). This process starts with expression of P-selectins and vascular cell adhesion molecule 1 (VCAM-1) by the inflamed brain endothelium. These proteins interact with ligands on circulating leukocytes, such as P-selectin glycoprotein ligand 1 (PSGL-1) and very late antigen 4 (VLA-4), to capture the cell. The leukocyte then rolls along the endothelium, causing activation of leukocytic integrins and enabling interaction with endothelial VCAM-1 and intracellular adhesion molecule 1 (ICAM-1), creating a firm adhesion. Next, the leukocyte crawls along the endothelium, mediated by chemokines expressed by endothelial cells, after which it enters the brain, either through an endothelial cell or via inter-endothelial junctions.

The entered immune cells release inflammatory factors such as cytokines, free radicals, and MMPs, which further exacerbate BBB disruption, either directly, or by activation of other cells of the NVU, such as astrocytes and microglia [26, 27, 29]. Immune cell infiltration into the brain is a hallmark of many common neurological diseases, including ischemic stroke [128], Alzheimer’s disease [129], Parkinson’s disease [130], and MS [131]. Several current therapies for MS are based on reducing immune cell entry into the brain. Monoclonal antibody drug Natalizumab blocks the interaction between VLA-4 on circulating immune cells and VCAM-1 on inflamed endothelium, inhibiting capture of immune cells and subsequent extravasation [132, 133]. Another commonly used drug, Fingolimod, prevents immune cell infiltration of the CNS by inducing internalization of sphingosine-1-phosphate receptors, sequestering lymphocytes to lymph nodes [134, 135]. The success of Natalizumab and Fingolimod resulted in the pursuit of novel therapies with similar mechanisms, but fewer side effects and improved pharmacokinetic properties. A recent example is posed by the FDA approval of Ozanimod in 2020, which resembles Fingolimod’s mode of action, but is more selective, causes fewer side effects, and shows shorter half-life [136]. While Natalizumab, Fingolimod, and Ozanimod are currently not included in standard treatments of stroke, Alzheimer’s disease and Parkinson’s disease, several studies did report beneficial effects for these indications, though via diverse mechanisms [137–139].

Addition of circulating immune cells to microfluidic NVU models allows researchers to study the cells’ mode of entrance into the brain and the mechanisms by which they exacerbate disease processes. Immune cells can be fluorescently labeled and perfused through the lumen of the NVU on-a-chip models and immune cell adhesion can be studied at baseline and after modeling disease, e.g. after cytokine addition to induce an inflammatory endothelial phenotype [140]. Subsequent immune cell extravasation and migration towards the CNS compartment of the chip can then be studied by tracking and quantifying the immune cells as presented in recent reports [141–143]. Furthermore, samples can be taken from apical and basolateral compartments of the chips – representing the blood and brain side, respectively – and cytokine contents can be analyzed, as shown in a study by Gijzen et al. [144].

### iPSC-derived models for personalized therapies

The cell types required to study the human NVU in vitro can be obtained from different sources. While it is challenging to obtain primary human material, the use of primary human brain endothelial cells, astrocytes, and pericytes is still common for microfluidic NVU modeling. A benefit of primary human cells is their fully differentiated state and physiological relevance – it is the actual material. However, it is known that primary cells can lose many of their characteristics when taken out of their in vivo environment, leading to loss of barrier function for brain endothelial cells or increased activation for glial cells [52, 145, 146]. Moreover, there is a logistical challenge since material from a single patient is limited, and donor variability can be significant. Immortalized cell lines generally offer a solution to this logistical challenge, allowing large banks of the same cell source to be generated and used for many experiments. Cell lines, however, are generally considered less physiologically relevant due to the modifications that are required to obtain the immortalized properties, displaying altered expression of TJ proteins, efflux transporters, and limited responsiveness to co-culture with supporting cells of the NVU [45, 53, 147]. In recent years, increasing focus has been placed on NVU cell sources obtained from stem cells, especially from iPSCs.

Current in vitro NVU models often make use of a mixture of primary, immortalized, and iPSC-derived cells from healthy donors. In most cases, the cells used within one model are not donor matched. Future models could incorporate donor-matched models of all-iPSC-derived cells obtained from healthy donors and donors carrying genetic risk factors for neurological disease. Recent work by Montagne and colleagues showed that *APOE4*, the major genetic risk factor of Alzheimer’s disease, causes BBB dysfunction that is predictive for cognitive decline, independently of Alzheimer’s disease pathology [148]. Genetic risk factors for other neurological diseases such as Parkinson’s disease, Huntington’s disease, and amyotrophic lateral sclerosis have also shown to result in BBB disruption. An extensive review on this topic is provided by Sweeney and colleagues [149]. Use of all-iPSC-derived models would allow the study of NVU function at single-patient level and enable assessment of personalized therapies for patients carrying genetic risk factors for neurological disease. It is important to note, however, that genetic risk factors only explain a small portion of all cases of neurological disease, and that discovery of therapeutic targets in risk-carrying patients often does not translate well to the general patient population.

Although the field of NVU modeling has placed major focus on iPSC-derived models in recent years [47, 150], the use of iPSC-derived cells comes with several limitations [151, 152]. First, the differentiation of iPSCs into the various cell types of the NVU is a laborious and costly process, as many cell types require several weeks or months of differentiation. Second, the resulting differentiated cells differ from their counterparts in vivo, for example in their level of maturity or overall phenotype, which is of concern especially with current protocols for differentiation of iPSC-derived brain endothelial cells [153]. Third, valuable features of cells may be lost after reprogramming to the iPSC stage, which complicates the modeling of patient phenotypes. In summary, iPSC-derived donor matched models may hold great potential for studying neurological disease and for personalized medicine applications in future NVU on-a-chip models but come with several limitations that may outweigh their advantages. This must be assessed on a case-by-case basis.

### Modeling neurological disease

Stroke is the second cause of death and a major cause of adult disability worldwide [1, 2, 154]. Of all stroke cases, approximately 80% is of ischemic nature, resulting from a thrombus impairing blood flow to the brain. As a result of the halted flow, the brain receives insufficient oxygen and nutrients, causing a detrimental cascade that involves BBB breakdown and neuronal cell death [155, 156]. We have previously modeled stroke using an NVU-on-a-chip model by mimicking hypoglycemic and hypoxic conditions – using glucose-free medium and chemical hypoxia, respectively – and by stopping medium perfusion. The resulting cultures showed several phenotypes observed in ischemic stroke, including impaired BBB integrity, lowered mitochondrial potential, and decreased ATP levels [101].

NVU on-a-chip models can also be employed to model other common neurological diseases. One route would involve the use of iPSC-derived cells from patients with a genetic risk factor, as discussed in the previous section. For Alzheimer’s disease, one could employ iPSC-derived NVU models from donors carrying the *APOE4* genotype [148]. Alternatively, Alzheimer’s disease can be modeled by exposing NVU-on-a-chip models to proteins involved in Alzheimer’s pathology, such as phosphorylated Tau, amyloid β (Aβ), or apolipoprotein E (APOE) [157]. Robert and colleagues added Aβ monomers to the basal side of a 3D NVU model and showed transport of the monomers to the lumen of the endothelial vessels [158]. In addition, the authors show that APOE4 is less effective than the protective APOE2 in promoting Aβ transport, in line with clinical findings. A review of NVU on-a-chip models for the study of Alzheimer’s disease is provided by Yoon et al. [159].

Similar approaches can be taken to study Parkinson’s disease in NVU-on-a-chip models. One could establish a patient-derived model employing iPSCs from patients with mutations commonly found in Parkinson’s disease, such as *LRRK2, PRKN*, *PINK1*, and *PRRK2* [160]. Recent work by De Rus Jacuet and colleagues describes co-culture of iPSC-derived brain endothelial-like cells and astrocytes derived from Parkinson’s patients carrying a mutation in LRRK2 in a microfluidic chip. The authors showed a role for inflammatory astrocytes in BBB leakage observed in Parkinson’s disease, which was attenuated by inhibition of mitogen-activated protein kinase kinase 1/2 (MEK1/2) signaling [161]. Alternatively, one could employ healthy cells and mimic Parkinson’s disease using proteins involved in Parkinson’s disease pathology, such as α-synuclein [162]. A study by Pediaditakis et al. showed that α-synuclein exposure of the brain side of an NVU-on-a-chip lead to reduced barrier function in the adjacent endothelial compartment [163].

To model NVU dysfunction in MS, accurate modeling of neuroinflammatory processes is required [164]. To this end, the NVU-on-a-chip models can be exposed to factors that weaken tight junctions, promote leukocyte adhesion and extravasation, and induce microglia activation. One option is to employ pro-inflammatory cytokines, such as IL-1β, IL-6, or TNFα [26], which cause weakening of tight junctions. Alternatively, chemokine motif ligands (CXCL) may be employed. Recent work by Nair et al. described the culture of primary human brain endothelial cells in a microfluidic chip and showed barrier disruption, endothelial inflammation, and T cell migration under neuroinflammatory conditions induced by the presence of TNFα, IL-1β, and CXCL12 [165]. Another option is exposure to pathogen-derived molecules, such as lipopolysaccharide (LPS), which has been extensively used to induce BBB permeability, promote production of cytokines, chemokines and MMPs, and activate microglia in neuroinflammation models [166–169]. These inflammation-inducing approaches combined with the incorporation of microglia and circulating immune cells, will allow for complex in vitro modeling of neuroinflammation in microfluidic NVU models.

### Heterogeneity of the neurovascular unit

**Fig. 5:**
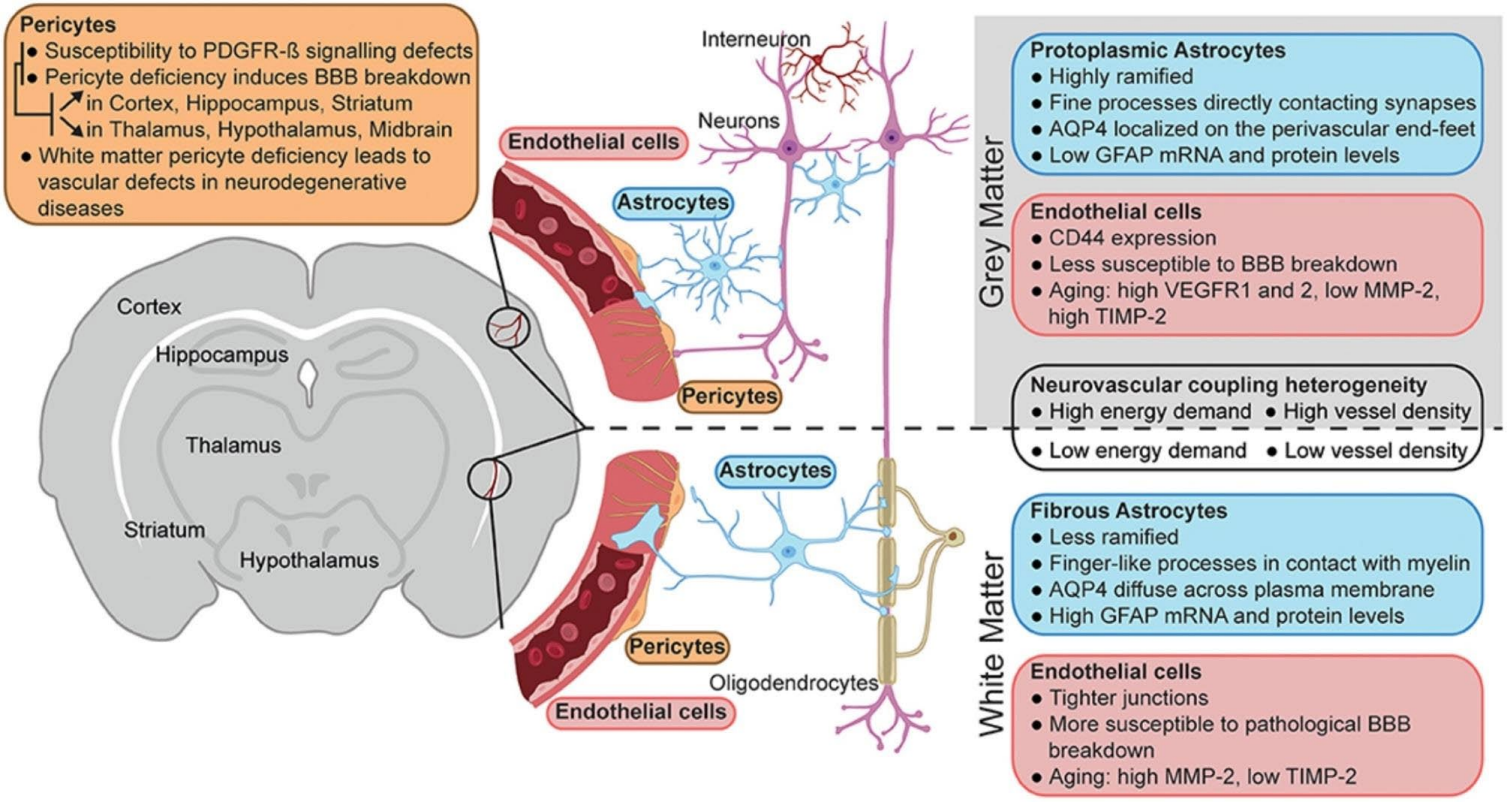
Neurovascular unit heterogeneity between gray and white matter. Schematic representation of the main differences between the neurovascular unit of gray (top) vs. white matter (bottom). The figure was created using Biorender and published by Bernier et al. (2021). The figure was reused in compliance with the requirements of the Creative Commons CC-BY license under which it was published

Many studies approach the NVU as a uniform structure. In contrast, the NVU is highly heterogeneous [7, 8, 21, 170]. The vessels that make up the NVU come in different diameters, which show differences in their relative permeability, transporter expression, and interaction with perivascular cells [171–173]. In addition, the NVU shows different characteristics in different regions of the CNS. Blood vessels near ventricular zones contain fenestrations, display discontinuous TJs, and are permeable [174, 175]. In contrast, blood vessels in the cortex are much tighter. Even within the cortex itself, differences are found in vessels, for example between grey matter and white matter tissue [8, 176–179] (Fig. 5). Distinct regions of the CNS are affected differently during aging and in neurological disorders [180, 181]. When modeling specific diseases, taking local characteristics of the NVU – such as vessel diameter and ratios between endothelial cells and different supporting cells – in the affected area into account may improve relevance of the obtained results. To our knowledge, the current NVU models are yet to account for the heterogeneity of the NVU.

### Circadian rhythms and aging

In addition to its heterogeneous nature, the NVU is also highly dynamic. Animal studies have shown that circadian rhythms influence the permeability of brain vasculature by dynamic regulation of efflux transporter P-glycoprotein (P-gp) [182, 183] or dynamic regulation of gap junction opening [184]. These circadian rhythms can be employed to target drugs into the brain more effectively by administering drugs at optimal times during the day. Recent reviews of circadian rhythms and the neurovascular unit are provided by Schurhoff & Toborek and Skapetze et al. [185, 186].

In the early 1970s, researchers first described the existence of a clock gene termed Period in Drosophila [184]. In following years, more clock genes and transcription factors were discovered, including but not limited to Clock [187], Bmal1 [188, 189], and Cry [190]. Together, these form a feedback loop that controls transcription and translation of proteins that are required for generation and regulation of circadian rhythms [191]. These clock genes were later discovered in many mammalian cells and essential for circadian rhythms.

To date, no in vitro NVU models have incorporated circadian rhythms to the best of our knowledge. Several methods have been reported to enable synchronization of circadian rhythms in in vitro cultures. One such method comes in the form of serum shock, in which cells are exposed to high concentrations of serum for a short period of time to synchronize circadian gene expression [192]. Another commonly used method employs analogues of cyclic adenosine monophosphate (cAMP), which is an indispensable component of the mammalian circadian clock [193]. Addition of cAMP analogues, such as forskolin, to the cell culture medium of NVU-on-a-chip models for a short period of time can synchronize circadian gene expression [194, 195]. Alternatively, one can engineer cells to express receptors for neuropeptides involved in circadian rhythms and expose cultures to those neuropeptides to induce synchronicity. This approach was successfully applied by Han and colleagues in a microfluidic chip incorporating fibroblasts [196]. Lastly, a circadian rhythm could be achieved in the NVU-on-a-chip models using temperature fluctuations, which were shown to synchronize circadian rhythms in vitro with improved success compared to chemical synchronizers in iPSCs [197, 198]. Given that circadian rhythms vary between cell types in vivo [183, 199], a temperature-based approach may also allow for more relevant synchronization than use of chemical synchronizers, which may reset the rhythms of different cell types to the same phase.

In addition to circadian rhythms, the NVU also shows longer-term dynamics. Recently, more focus has been placed on the changes that occur in the NVU with aging. With aging, substantial changes occur in all cellular and non-cellular components of the NVU [200, 201]. The aging NVU displays increased oxidative stress, weakening of tight junctions, reduced interaction between vascular and perivascular cells, and diminished clearance of toxic molecules from the brain. Current and future microfluidic NVU models can be employed to study age related changes in the NVU, which may lead to new insights on how to preserve NVU function in aging individuals. An extensive review by Osipova and colleagues suggested five approaches for inducing an aged phenotype in in vitro models of the NVU [202]. The first approach constitutes induction of a senescence-associated secretory phenotype [203] and inflammasome activation [204]. A second approach focuses on induction of insulin resistance, which is associated with aging in human cells and was reported to be linked to pathologies in Alzheimer’s disease [205, 206]. A third option considers the manipulation of nicotinamide adenine dinucleotide (NAD+), as a decrease in NAD + is observed in aging and replenishing NAD + levels may improve cell lifespan via DNA repair and mitochondrial maintenance [207]. The fourth approach involves induction of DNA damage, for example via exposure to gamma radiation [208], and a subsequent DNA damage response (DDR). As a fifth option, the authors suggested promotion of mitochondrial biogenesis [209], glycolytic changes [208, 210], and increased production of lactate [211].

Another possibility lies in progerin-induced aging. Progeria is a rare disease in which individuals age rapidly, due to a mutation in the gene lamin A, resulting in a shorter transcript known as progerin. The mutation causes aberrant chromatin organization, DNA damage response, and cell cycle and telomerase function, leading to premature aging and cellular senescence [212]. Overexpression of progerin in iPSC-derived cells is of use in modeling neurogenerative diseases, as was shown by Miller and colleagues, who found relevant disease phenotypes in a progerin-induced aged model of Parkinson’s disease [213].

In addition to the aforementioned approaches, which focus on the induction of an age-related phenotype, it is also possible to model aging in the NVU by utilizing cells derived from older individuals. An example is provided by Galatro et al., who studied gene expression in microglia obtained from postmortem material of donors ranging from the age of 34 to 102 [214]. The authors found that the alterations in gene expression observed with aging included many actin cytoskeleton-associated genes and genes involved in cell adhesion, axonal guidance, and the sensome.

## Conclusions

The introduction of microfluidic cell culture platforms has led to tremendous progress in the field of NVU modeling. Microfluidic NVU models show increased complexity compared to traditional models, allowing for co-culture of various cell types, incorporation of cell-matrix interactions, and presence of fluid flow. Furthermore, the recent introduction of microfluidic chips in higher throughput formats now renders NVU on-a-chip models compatible with routine laboratory adoption and assessment of novel drug candidates.

With time, NVU on-a-chip models have shown increasing biological complexity. More emphasis is placed on the use of primary cells and iPSC-derived cells which allow more accurate disease- and patient-specific models. Further improvements in protocols for cell differentiation and continued incorporation and characterization of immune cells, both resident and circulating, will improve future NVU models’ relevance even further. Lastly, NVU models can be adapted to account for circadian rhythms, changes observed in aging, and the heterogeneity of the NVU.

Importantly, the NVU is a highly complex structure, and it is likely that no model will be able to capture all its features. Fit-for-purpose models provide a viable compromise between physiological relevance and ease-of-use and hold the future of NVU modeling: as simple as possible, as complex as needed.

## Data Availability

Not applicable.

## Abbreviations

2D: two-dimensional
3D: three-dimensional:AAV:adeno-associated virus
Aβ: amyloid beta
AJ: adherens junction
APOE: apolipoprotein E
BBB: blood-brain barrier
BCRP: breast cancer resistance protein
BEC: brain endothelial cell
cAMP: cyclic adenosine monophosphate
CMT: carrier-mediated transport
CNS: central nervous system
CXCL: chemokine motif ligand
DDR: DNA damage response
GLUT: glucose transporter
HUVEC: human umbilical vein endothelial cell
IL: interleukin
INSR: insulin receptor
LAT: L-type amino acid transporter
LDLR: low-density lipoprotein receptor
LPS: lipopolysaccharide
LRPR: LDL-related protein receptors
MEK1: mitogen-activated protein kinase kinase
MMP: matrix metalloproteinases
MRP: multidrug resistance protein
MS: multiple sclerosis
NAD+: nicotinamide adenine dinucleotide
NVU: neurovascular unit
ICAM: intracellular adhesion molecule
iPSC: induced pluripotent stem cell
PDMS: polydimethylsiloxane
P-gp: P-glycoprotein
PSGL: P-selectin glycoprotein ligand
RMT: receptor mediated transcytosis
TJ: tight junction
TNF: tumor necrosis factor
VCAM: vascular cell adhesion molecule
VLA: very late antigen

## References

[1] Donkor ES (2018). Stroke in the 21st Century: a snapshot of the Burden, Epidemiology, and Quality of Life. Stroke Res Treat.

[2] Global Health Estimates. 2020: Deaths by Cause, Age, Sex, by Country and by Region, 2000–2019. Geneva, World Health Organization; 2020.

[3] Kuźma E, Lourida I, Moore SF, Levine DA, Ukoumunne OC, Llewellyn DJ (2018). Stroke and Dementia risk: a systematic review and meta-analysis. Alzheimers Dement.

[4] Craig L, Hoo ZL, Yan TZ, Wardlaw J, Quinn TJ (2022). Prevalence of Dementia in ischaemic or mixed Stroke populations: systematic review and meta-analysis. J Neurol Neurosurg Psychiatry.

[5] Azevedo FAC, Carvalho LRB, Grinberg LT, Farfel JM, Ferretti REL, Leite REP (2009). Equal numbers of neuronal and nonneuronal cells make the human brain an isometrically scaled-up primate brain. J Comp Neurol.

[6] Begley DJ, Brightman MW, Jucker E (2003). Structural and functional aspects of the blood-brain barrier. Progress in Drug Research.

[7] Wilhelm I, Nyúl-Tóth Á, Suciu M, Hermenean A, Krizbai IA (2016). Heterogeneity of the blood-brain barrier. Tissue Barriers.

[8] Noumbissi ME, Galasso B, Stins MF (2018). Brain vascular heterogeneity: implications for Disease pathogenesis and design of in vitro blood-brain barrier models. Fluids Barriers CNS.

[9] Matias I, Morgado J, Gomes FCA (2019). Astrocyte heterogeneity: impact to Brain Aging and Disease. Front Aging Neurosci.

[10] Tan YL, Yuan Y, Tian L (2020). Microglial regional heterogeneity and its role in the brain. Mol Psychiatry.

[11] Spector R (2009). Nutrient transport systems in brain: 40 years of progress. J Neurochem.

[12] Kadry H, Noorani B, Cucullo L (2020). A blood-brain barrier overview on structure, function, impairment, and biomarkers of integrity. Fluids Barriers CNS.

[13] Shen S, Zhang W (2010). ABC transporters and drug efflux at the blood-brain barrier. Rev Neurosci.

[14] Taggi V, Romo MR, Piquette-miller M, Zu Schwabedissen HEM, Neuhoff S. Transporter regulation in critical protective barriers: Focus on Brain and Placenta. Pharmaceutics. 2022;14(7). 10.3390/pharmaceutics14071376 PMC9319476 35890272

[15] Wolburg H, Lippoldt A (2002). Tight junctions of the blood-brain barrier: development, composition and regulation. Vascul Pharmacol.

[16] Tietz S, Engelhardt B (2015). Brain barriers: crosstalk between complex tight junctions and adherens junctions. J Cell Biol.

[17] Abbott NJ, Rönnbäck L, Hansson E (2006). Astrocyte–endothelial interactions at the blood–brain barrier. Nat Rev Neurosci 2006 71.

[18] Sá-Pereira I, Brites D, Brito MA (2012). Neurovascular unit: a focus on pericytes. Mol Neurobiol.

[19] Bell AH, Miller SL, Castillo-Melendez M, Malhotra A (2019). The neurovascular unit: effects of Brain insults during the Perinatal Period. Front Neurosci.

[20] Daneman R (2012). The blood-brain barrier in health and Disease. Ann Neurol.

[21] Profaci CP, Munji RN, Pulido RS, Daneman R. The blood-brain barrier in health and Disease: important unanswered questions. J Exp Med. 2020;217(4). 10.1084/jem.20190062 PMC7144528 32211826

[22] Ronaldson PT, Davis TP (2020). Regulation of blood-brain barrier integrity by microglia in health and Disease: a therapeutic opportunity. J Cereb Blood Flow Metab.

[23] Engelhardt B (2008). Immune cell entry into the central nervous system: involvement of adhesion molecules and chemokines. J Neurol Sci.

[24] Engelhardt B, Ransohoff RM (2012). Capture, crawl, cross: the T cell code to breach the blood-brain barriers. Trends Immunol.

[25] Marchetti L, Engelhardt B (2020). Immune cell trafficking across the blood-brain barrier in the absence and presence of neuroinflammation. Vasc Biol.

[26] De Vries HE, Blom-Roosemalen MCM, Van Oosten M, De Boer AG, Van Berkel TJC, Breimer DD (1996). The influence of cytokines on the integrity of the blood-brain barrier in vitro. J Neuroimmunol.

[27] Lee DH, Gold R, Linker RA (2012). Mechanisms of oxidative damage in multiple sclerosis and neurodegenerative Diseases: therapeutic modulation via Fumaric Acid Esters. Int J Mol Sci.

[28] Pun PBL, Lu J, Moochhala S (2009). Involvement of ROS in BBB dysfunction. Free Radic Res.

[29] Lakhan SE, Kirchgessner A, Tepper D, Leonard A (2013). Matrix metalloproteinases and blood-brain barrier disruption in acute ischemic Stroke. Front Neurol.

[30] Pardridge WM (2012). Drug transport across the blood–brain barrier. J Cereb Blood Flow Metab.

[31] Pardridge WM. A historical review of Brain Drug Delivery. Pharmaceutics. 2022;14(6). 10.3390/pharmaceutics14061283 PMC9229021 35745855

[32] Hersh DS, Wadajkar AS, Roberts N, Perez JG, Connolly NP, Frenkel V (2016). Evolving drug delivery strategies to overcome the blood brain barrier. Curr Pharm Des.

[33] Dong X (2018). Current strategies for Brain Drug Delivery. Theranostics.

[34] Syvänen S, Lindhe Ö, Palner M, Kornum BR, Rahman O, Långström B (2009). Species differences in blood-brain barrier transport of three positron emission tomography radioligands with emphasis on P-glycoprotein transport. Drug Metab Dispos.

[35] Nicolas J-M. Species differences and Impact of Disease State on BBB. In: Di L, Kerns EH, editors. Blood-brain barrier in Drug Discovery. Wiley; 2015. pp. 66–93.

[36] O’Brown NM, Pfau SJ, Gu C (2018). Bridging barriers: a comparative look at the blood-brain barrier across organisms. Genes Dev.

[37] Bhalerao A, Sivandzade F, Archie SR, Chowdhury EA, Noorani B, Cucullo L. In vitro modeling of the neurovascular unit: advances in the field. Fluids Barriers CNS. 2020;17(1). 10.1186/s12987-020-00183-7 PMC7077137 32178700

[38] Joó F, Karnushina I (1973). A procedure for the isolation of capillaries from rat brain. Cytobios.

[39] DeBault LE, Cancilla PA (1980). γ-glutamyl transpeptidase in isolated brain endothelial cells: induction by glial cells in vitro. Science.

[40] Tao-Cheng JH, Nagy Z, Brightman MW (1987). Tight junctions of brain endothelium in vitro are enhanced by astroglia. J Neurosci.

[41] Mischeck U, Meyer J, Galla HJ (1989). Characterization of γ-glutamyl transpeptidase activity of cultured endothelial cells from porcine brain capillaries. Cell Tissue Res.

[42] Dehouck M-P, Méresse S, Delorme P, Fruchart J, ‐C, Cecchelli R (1990). An easier, reproducible, and Mass‐Production Method to study the blood–brain barrier in Vitro. J Neurochem.

[43] Hayashi Y, Nomura M, Yamagishi S, Harada S, Yamashita J, Yamamoto H (1997). Induction of various blood-brain Barrier properties in Non-neural endothelial cells by close apposition to co-cultured astrocytes. Glia.

[44] Hayashi K, Nakao S, Nakaoke R, Nakagawa S, Kitagawa N, Niwa M (2004). Effects of hypoxia on endothelial/pericytic co-culture model of the blood-brain barrier. Regul Pept.

[45] Weksler BB, Subileau EA, Perriere N, Charneau P, Holloway K, Leveque M (2005). Blood-brain barrier-specific properties of a human adult brain endothelial cell line. Faseb J.

[46] Takeshita Y, Obermeier B, Cotleur A, Sano Y, Kanda T, Ransohoff RM (2014). An in vitro blood–brain barrier model combining shear stress and endothelial cell/astrocyte co-culture. J Neurosci Methods.

[47] Lippmann ES, Azarin SM, Kay JE, Nessler RA, Wilson HK, Al-Ahmad A (2012). Derivation of blood-brain barrier endothelial cells from human pluripotent stem cells. Nat Biotechnol.

[48] Cecchelli R, Aday S, Sevin E, Almeida C, Culot M, Dehouck L (2014). A stable and reproducible human blood-brain barrier model derived from hematopoietic stem cells. PLoS ONE.

[49] Urich E, Patsch C, Aigner S, Graf M, Iacone R, Freskgård PO (2013). Multicellular self-assembled spheroidal model of the blood brain barrier. Sci Rep.

[50] Cho C-F, Wolfe JM, Fadzen CM, Calligaris D, Hornburg K, Chiocca EA (2017). Blood-brain-barrier spheroids as an in vitro screening platform for brain-penetrating agents. Nat Commun.

[51] Simonneau C, Duschmalé M, Gavrilov A, Brandenberg N, Hoehnel S, Ceroni C (2021). Investigating receptor-mediated antibody transcytosis using blood-brain barrier organoid arrays. Fluids Barriers CNS.

[52] Helms HC, Abbott NJ, Burek M, Cecchelli R, Couraud PO, Deli MA (2016). In vitro models of the blood–brain barrier: an overview of commonly used brain endothelial cell culture models and guidelines for their use. J Cereb Blood Flow Metab.

[53] Sivandzade F, Cucullo L (2018). In-vitro blood-brain barrier modeling: a review of modern and fast-advancing technologies. J Cereb Blood Flow Metab.

[54] Hoffman RM (1993). To do tissue culture in two or three dimensions? That is the question. Stem Cells.

[55] Abbott NJ, Hughes CC, Revest PA, Greenwood J (1992). Development and characterisation of a rat brain capillary endothelial culture: towards an in vitro blood-brain barrier. J Cell Sci.

[56] van Duinen V, Trietsch SJ, Joore J, Vulto P, Hankemeier T (2015). Microfluidic 3D cell culture: from tools to tissue models. Curr Opin Biotechnol.

[57] Stanness KA, Guatteo E, Janigro D (1996). A dynamic model of the blood-brain barrier in vitro. Neurotoxicology.

[58] Cucullo L, McAllister MS, Kight K, Krizanac-Bengez L, Marroni M, Mayberg MR (2002). A new dynamic in vitro model for the multidimensional study of astrocyte-endothelial cell interactions at the blood-brain barrier. Brain Res.

[59] Neuhaus W, Lauer R, Oelzant S, Fringeli UP, Ecker GF, Noe CR (2006). A novel flow based hollow-fiber blood-brain barrier in vitro model with immortalised cell line PBMEC/C1-2. J Biotechnol.

[60] Booth R, Kim H (2012). Characterization of a microfluidic in vitro model of the blood-brain barrier (µBBB). Lab Chip.

[61] Griep LM, Wolbers F, De Wagenaar B, Ter Braak PM, Weksler BB, Romero IA (2013). BBB on CHIP: microfluidic platform to mechanically and biochemically modulate blood-brain barrier function. Biomed Microdevices.

[62] Prabhakarpandian B, Shen M-C, Nichols JB, Mills IR, Sidoryk-Wegrzynowicz M, Aschner M (2013). SyM-BBB: a microfluidic blood brain barrier model. Lab Chip.

[63] Achyuta AKH, Conway AJ, Crouse RB, Bannister EC, Lee RN, Katnik CP (2013). A modular approach to create a neurovascular unit-on-a-chip. Lab Chip.

[64] Cho H, Seo JH, Wong KHK, Terasaki Y, Park J, Bong K (2015). Three-dimensional blood-brain barrier model for in vitro studies of neurovascular pathology. Sci Rep.

[65] Sellgren KL, Hawkins BT, Grego S. An optically transparent membrane supports shear stress studies in a three-dimensional microfluidic neurovascular unit model. Biomicrofluidics. 2015;9(6). 10.1063/1.4935594 PMC4644144 26594261

[66] Herland A, Van Der Meer AD, FitzGerald EA, Park TE, Sleeboom JJF, Ingber DE (2016). Distinct contributions of astrocytes and pericytes to neuroinflammation identified in a 3D human blood-brain barrier on a chip. PLoS ONE.

[67] Walter FR, Valkai S, Kincses A, Petneházi A, Czeller T, Veszelka S (2016). A versatile lab-on-a-chip tool for modeling biological barriers. Sens Actuators B Chem.

[68] Adriani G, Ma D, Pavesi A, Kamm RD, Goh ELK (2017). A 3D neurovascular microfluidic model consisting of neurons, astrocytes and cerebral endothelial cells as a blood-brain barrier. Lab Chip.

[69] Brown JA, Pensabene V, Markov DA, Allwardt V, Diana Neely M, Shi M et al. Recreating blood-brain barrier physiology and structure on chip: a novel neurovascular microfluidic bioreactor. Biomicrofluidics. 2015;9(5). 10.1063/1.4934713 PMC4627929 26576206

[70] Vatine GD, Barrile R, Workman MJ, Sances S, Barriga BK, Rahnama M (2019). Human iPSC-Derived blood-brain barrier chips enable Disease modeling and Personalized Medicine Applications. Cell Stem Cell.

[71] Park TE, Mustafaoglu N, Herland A, Hasselkus R, Mannix R, FitzGerald EA (2019). Hypoxia-enhanced blood-brain barrier chip recapitulates human barrier function and shuttling of Drugs and antibodies. Nat Commun.

[72] Leung CM, de Haan P, Ronaldson-Bouchard K, Kim GA, Ko J, Rho HS et al. A guide to the organ-on-a-chip. Nat Rev Methods Prim 2022 21. 2022;2(1):1–29.

[73] Ingber DE (2022). Human organs-on-chips for Disease modelling, drug development and personalized medicine. Nat Rev Genet.

[74] Singh D, Mathur A, Arora S, Roy S, Mahindroo N (2022). Journey of organ on a chip technology and its role in future healthcare scenario. Appl Surf Sci Adv.

[75] Ng JMK, Gitlin I, Stroock AD, Whitesides GM (2002). Components for integrated poly(dimethylsiloxane) microfluidic systems. Electrophoresis.

[76] Campbell SB, Wu Q, Yazbeck J, Liu C, Okhovatian S, Radisic M (2021). Beyond polydimethylsiloxane: alternative materials for fabrication of Organ-on-a-Chip devices and Microphysiological systems. ACS Biomater Sci Eng.

[77] Shakeri A, Khan S, Didar TF (2021). Conventional and emerging strategies for the fabrication and functionalization of PDMS-based microfluidic devices. Lab Chip.

[78] Wong I, Ho C-M (2009). Surface molecular property modifications for poly(dimethylsiloxane) (PDMS) based microfluidic devices. Microfluid Nanofluidics.

[79] Zhang H, Chiao M (2015). Anti-fouling Coatings of Poly(dimethylsiloxane) devices for Biological and Biomedical Applications. J Med Biol Eng.

[80] Toepke MW, Beebe DJ (2006). PDMS absorption of small molecules and consequences in microfluidic applications. Lab Chip.

[81] Regehr KJ, Domenech M, Koepsel JT, Carver KC, Ellison-Zelski SJ, Murphy WL (2009). Biological implications of polydimethylsiloxane-based microfluidic cell culture. Lab Chip.

[82] Wang JD, Douville NJ, Takayama S, Elsayed M (2012). Quantitative analysis of molecular absorption into PDMS microfluidic channels. Ann Biomed Eng.

[83] Shirure VS, George SC (2017). Design considerations to minimize the impact of drug absorption in polymer-based organ-on-a-chip platforms. Lab Chip.

[84] van Meer BJ, de Vries H, Firth KSA, van Weerd J, Tertoolen LGJ, Karperien HBJ (2017). Small molecule absorption by PDMS in the context of drug response bioassays. Biochem Biophys Res Commun.

[85] Probst C, Schneider S, Loskill P (2018). High-throughput organ-on-a-chip systems: current status and remaining challenges. Curr Opin Biomed Eng.

[86] Hudecz D, McCloskey MC, Vergo S, Christensen S, McGrath JL, Nielsen MS (2023). Modelling a human blood-brain barrier co-culture using an ultrathin Silicon Nitride membrane-based microfluidic device. Int J Mol Sci.

[87] Kawakita S, Mandal K, Mou L, Mecwan MM, Zhu Y, Li S (2022). Organ-on-a-Chip models of the blood-brain barrier: recent advances and future prospects. Small.

[88] Cai Y, Fan K, Lin J, Ma L, Li F (2022). Advances in BBB on Chip and Application for studying reversible opening of blood-brain barrier by Sonoporation. Micromachines.

[89] Guarino V, Zizzari A, Bianco M, Gigli G, Moroni L, Arima V. Advancements in modelling human blood brain-barrier on a chip. Biofabrication. 2023;15(2). 10.1088/1758-5090/acb571 36689766

[90] Vargas R, Egurbide-Sifre A, Medina L (2021). Organ-on-a-Chip systems for new Drugs development. ADMET DMPK.

[91] Trietsch SJ, Israëls GD, Joore J, Hankemeier T, Vulto P (2013). Microfluidic titer plate for stratified 3D cell culture. Lab Chip.

[92] Soragni C, Queiroz K, Ng CP, Stok A, Olivier T, Tzagkaraki D et al. Phenotypic screening in Organ-on-a-Chip systems: a 1537 kinase inhibitor library screen on a 3D angiogenesis assay. Angiogenesis. 2023;1–13. 10.1007/s10456-023-09888-3 PMC10881651 37493987

[93] Fantini S, Sassaroli A, Tgavalekos KT, Kornbluth J (2016). Cerebral blood flow and autoregulation: current measurement techniques and prospects for noninvasive optical methods. Neurophotonics.

[94] Nippert AR, Biesecker KR, Newman EA (2018). Mechanisms mediating functional hyperemia in the brain. Neuroscientist.

[95] Reinitz A, DeStefano J, Ye M, Wong AD, Searson PC (2015). Human brain microvascular endothelial cells resist elongation due to shear stress. Microvasc Res.

[96] DeStefano JG, Xu ZS, Williams AJ, Yimam N, Searson PC (2017). Effect of shear stress on iPSC-derived human brain microvascular endothelial cells (dhBMECs). Fluids Barriers CNS.

[97] Seebach J, Dieterich P, Luo F, Schillers H, Vestweber D, Oberleithner H (2000). Endothelial barrier function under laminar fluid shear stress. Lab Invest.

[98] Siddharthan V, Kim YV, Liu S, Kim KS (2007). Human astrocytes/astrocyte-conditioned medium and shear stress enhance the barrier properties of human brain microvascular endothelial cells. Brain Res.

[99] Cucullo L, Hossain M, Puvenna V, Marchi N, Janigro D (2011). The role of shear stress in blood-brain barrier endothelial physiology. BMC Neurosci.

[100] Wevers NR, Kasi DG, Gray T, Wilschut KJ, Smith B, van Vught R (2018). A perfused human blood–brain barrier on-a-chip for high-throughput assessment of barrier function and antibody transport. Fluids Barriers CNS.

[101] Wevers NR, Nair AL, Fowke TM, Pontier M, Kasi DG, Spijkers XM (2021). Modeling ischemic Stroke in a triculture neurovascular unit on-a-chip. Fluids Barriers CNS.

[102] Dewey CF, Bussolari SR, Gimbrone MA, Davies PF (1981). The dynamic response of vascular endothelial cells to fluid shear stress. J Biomech Eng.

[103] Cucullo L, Hossain M, Tierney W, Janigro D (2013). A new dynamic in vitro modular capillaries-venules modular system: cerebrovascular physiology in a box. BMC Neurosci.

[104] Chiu JJ, Chien S (2011). Effects of disturbed flow on vascular endothelium: pathophysiological basis and clinical perspectives. Physiol Rev.

[105] Xanthis I, Souilhol C, Serbanovic-Canic J, Roddie H, Kalli AC, Fragiadaki M (2019). β1 integrin is a sensor of blood flow direction. J Cell Sci.

[106] Ahn SI, Sei YJ, Park HJ, Kim J, Ryu Y, Choi JJ (2020). Microengineered human blood–brain barrier platform for understanding nanoparticle transport mechanisms. Nat Commun 2020 111.

[107] Goldmann T, Wieghofer P, Jordão MJC, Prutek F, Hagemeyer N, Frenzel K (2016). Origin, fate and dynamics of macrophages at central nervous system interfaces. Nat Immunol.

[108] Masuda T, Prinz M, Microglia (2016). A unique versatile cell in the Central Nervous System. ACS Chem Neurosci.

[109] Schebesch C, Kodelja V, Müller C, Hakij N, Bisson S, Orfanos CE (1997). Alternatively activated macrophages actively inhibit proliferation of peripheral blood lymphocytes and CD4 + T cells in vitro. Immunology.

[110] Franco R, Fernández-Suárez D (2015). Alternatively activated microglia and macrophages in the central nervous system. Prog Neurobiol.

[111] Chiu IM, Morimoto ETA, Goodarzi H, Liao JT, O’Keeffe S, Phatnani HP (2013). A neurodegeneration-specific gene-expression signature of acutely isolated microglia from an Amyotrophic Lateral Sclerosis mouse model. Cell Rep.

[112] Kan MJ, Lee JE, Wilson JG, Everhart AL, Brown CM, Hoofnagle AN (2015). Arginine deprivation and immune suppression in a mouse model of Alzheimer’s Disease. J Neurosci.

[113] Fantin A, Vieira JM, Gestri G, Denti L, Schwarz Q, Prykhozhij S (2010). Tissue macrophages act as cellular chaperones for vascular anastomosis downstream of VEGF-mediated endothelial tip cell induction. Blood.

[114] Haruwaka K, Ikegami A, Tachibana Y, Ohno N, Konishi H, Hashimoto A (2019). Dual microglia effects on blood brain barrier permeability induced by systemic inflammation. Nat Commun.

[115] Thurgur H, Pinteaux E (2019). Microglia in the neurovascular unit: blood-brain barrier-microglia interactions after Central Nervous System disorders. Neuroscience.

[116] Jolivel V, Bicker F, Binamé F, Ploen R, Keller S, Gollan R (2015). Perivascular microglia promote blood vessel disintegration in the ischemic penumbra. Acta Neuropathol.

[117] Sumi N, Nishioku T, Takata F, Matsumoto J, Watanabe T, Shuto H (2010). Lipopolysaccharide-activated microglia induce dysfunction of the blood-brain barrier in rat microvascular endothelial cells co-cultured with microglia. Cell Mol Neurobiol.

[118] Kangwantas K, Pinteaux E, Penny J (2016). The extracellular matrix protein laminin-10 promotes blood-brain barrier repair after hypoxia and inflammation in vitro. J Neuroinflammation.

[119] Krasnow SM, Knoll JG, Verghese SC, Levasseur PR, Marks DL (2017). Amplification and propagation of interleukin-1β signaling by murine brain endothelial and glial cells. J Neuroinflammation.

[120] Fiala M, Looney DJ, Stins M, Way DD, Zhang L, Gan X (1997). TNF-α opens a Paracellular Route for HIV-1 Invasion across the blood-brain barrier. Mol Med.

[121] Nishioku T, Matsumoto J, Dohgu S, Sumi N, Miyao K, Takata F (2010). Tumor necrosis factor-α mediates the blood-brain barrier dysfunction induced by activated microglia in mouse brain microvascular endothelial cells. J Pharmacol Sci.

[122] Truettner JS, Alonso OF, Dietrich WD (2005). Influence of therapeutic Hypothermia on matrix metalloproteinase activity after traumatic brain injury in rats. J Cereb Blood Flow Metab.

[123] Yang Y, Estrada EY, Thompson JF, Liu W, Rosenberg GA (2007). Matrix metalloproteinase-mediated disruption of tight junction proteins in cerebral vessels is reversed by synthetic matrix metalloproteinase inhibitor in focal ischemia in rat. J Cereb Blood Flow Metab.

[124] Lyu Z, Park J, Kim K-M, Jin H-J, Wu H, Rajadas J (2021). A neurovascular-unit-on-a-chip for the evaluation of the restorative potential of stem cell therapies for ischaemic Stroke. Nat Biomed Eng 2021 58.

[125] Pediaditakis I, Kodella KR, Manatakis DV, Le CY, Barthakur S, Sorets A et al. A microengineered brain-chip to model neuroinflammation in humans. iScience. 2022;25(8). 10.1016/j.isci.2022.104813 PMC9379671 35982785

[126] Speicher AM, Wiendl H, Meuth SG, Pawlowski M (2019). Generating microglia from human pluripotent stem cells: novel in vitro models for the study of neurodegeneration. Mol Neurodegener.

[127] Wevers NR, de Vries HE (2016). Morphogens and blood-brain barrier function in health and Disease. Tissue Barriers.

[128] Planas AM (2018). Role of Immune cells migrating to the ischemic brain. Stroke.

[129] Ní Chasaide C, Lynch MA (2020). The role of the immune system in driving neuroinflammation. Brain Neurosci Adv.

[130] Kustrimovic N, Marino F, Cosentino M (2018). Peripheral immunity, Immunoaging and Neuroinflammation in Parkinson’s Disease. Curr Med Chem.

[131] Lopes Pinheiro MA, Kooij G, Mizee MR, Kamermans A, Enzmann G, Lyck R (2016). Immune cell trafficking across the barriers of the central nervous system in multiple sclerosis and Stroke. Biochim Biophys Acta.

[132] Leonard JP, Waldburger KE, Goldman SJ (1995). Prevention of experimental autoimmune encephalomyelitis by antibodies against interleukin 12. J Exp Med.

[133] Steinman L (2005). Blocking adhesion molecules as therapy for multiple sclerosis: natalizumab. Nat Rev Drug Discov.

[134] Mandala S, Hajdu R, Bergstrom J, Quackenbush E, Xie J, Milligan J (2002). Alteration of lymphocyte trafficking by sphingosine-1-phosphate receptor agonists. Science.

[135] Brinkmann V, Billich A, Baumruker T, Heining P, Schmouder R, Francis G (2010). Fingolimod (FTY720): discovery and development of an oral drug to treat multiple sclerosis. Nat Rev Drug Discov.

[136] Scott FL, Clemons B, Brooks J, Brahmachary E, Powell R, Dedman H (2016). Ozanimod (RPC1063) is a potent sphingosine-1-phosphate receptor-1 (S1P1) and receptor-5 (S1P5) agonist with autoimmune disease-modifying activity. Br J Pharmacol.

[137] Wendt TS, Li YJ, Liu Q, Shi F-D, Gonzales RJ (2020). Ozanimod attenuates Ischemia-Induced inflammation and dysfunction in human brain microvascular endothelial cells. FASEB J.

[138] Angelopoulou E, Piperi C (2019). Beneficial effects of Fingolimod in Alzheimer’s Disease: Molecular mechanisms and therapeutic potential. Neuromolecular Med.

[139] Zhao P, Yang X, Yang L, Li M, Wood K, Liu Q (2017). Neuroprotective effects of fingolimod in mouse models of Parkinson’s Disease. FASEB J.

[140] Poussin C, Kramer B, Lanz HL, van den Heuvel A, Laurent A, Olivier T (2020). 3D human microvessel-on-a-chip model for studying monocyte-to-endothelium adhesion under flow - application in systems toxicology. Altex.

[141] Gjorevski N, Avignon B, Gérard R, Cabon L, Roth AB, Bscheider M (2020). Neutrophilic infiltration in organ-on-a-chip model of tissue inflammation. Lab Chip.

[142] de Haan L, Suijker J, van Roey R, Berges N, Petrova E, Queiroz K (2021). A microfluidic 3D endothelium-on-a-Chip model to Study Transendothelial Migration of T Cells in Health and Disease. Int J Mol Sci.

[143] Ehlers H, Nicolas A, Schavemaker F, Heijmans JPM, Bulst M, Trietsch SJ (2023). Vascular inflammation on a chip: a scalable platform for trans-endothelial electrical resistance and immune cell migration. Front Immunol.

[144] Gijzen L, Marescotti D, Raineri E, Nicolas A, Lanz HL, Guerrera D (2020). An intestine-on-a-Chip model of Plug-and-play modularity to study inflammatory processes. SLAS Technol.

[145] Gumbleton M, Audus KL (2001). Progress and limitations in the use of in vitro cell cultures to serve as a permeability screen for the blood-brain barrier. J Pharm Sci.

[146] Lattke M, Guillemot F (2022). Understanding astrocyte differentiation: clinical relevance, technical challenges, and new opportunities in the omics era. Wires Mech Dis.

[147] Eigenmann DE, Xue G, Kim KS, Moses AV, Hamburger M, Oufir M (2013). Comparative study of four immortalized human brain capillary endothelial cell lines, hCMEC/D3, hBMEC, TY10, and BB19, and optimization of culture conditions, for an in vitro blood-brain barrier model for drug permeability studies. Fluids Barriers CNS.

[148] Montagne A, Nation DA, Sagare AP, Barisano G, Sweeney MD, Chakhoyan A (2020). APOE4 leads to blood–brain barrier dysfunction predicting cognitive decline. Nature.

[149] Sweeney MD, Zhao Z, Montagne A, Nelson AR, Zlokovic BV (2019). Blood-brain barrier: from physiology to Disease and back. Physiol Rev.

[150] Workman MJ, Svendsen CN (2020). Recent advances in human iPSC-derived models of the blood-brain barrier. Fluids Barriers CNS.

[151] Li L, Chao J, Shi Y (2018). Modeling neurological Diseases using iPSC-derived neural cells: iPSC modeling of neurological Diseases. Cell Tissue Res.

[152] Doss MX, Sachinidis A (2019). Current challenges of iPSC-Based Disease modeling and therapeutic implications. Cells.

[153] Lu TM, Houghton S, Magdeldin T, Durán JGB, Minotti AP, Snead A (2021). Pluripotent stem cell-derived epithelium misidentified as brain microvascular endothelium requires ETS factors to acquire vascular fate. Proc Natl Acad Sci.

[154] Feigin VL, Nichols E, Alam T, Bannick MS, Beghi E, Blake N (2019). Global, regional, and national burden of neurological disorders, 1990–2016: a systematic analysis for the global burden of Disease Study 2016. Lancet Neurol.

[155] Xing C, Arai K, Lo EH, Hommel M (2012). Pathophysiologic cascades in ischemic Stroke. Int J Stroke.

[156] Jayaraj RL, Azimullah S, Beiram R, Jalal FY, Rosenberg GA (2019). Neuroinflammation: friend and foe for ischemic Stroke. J Neuroinflammation.

[157] Long JM, Holtzman DM (2019). Alzheimer Disease: an update on pathobiology and treatment strategies. Cell.

[158] Robert J, Button EB, Yuen B, Gilmour M, Kang K, Bahrabadi A (2017). Clearance of beta-amyloid is facilitated by apolipoprotein E and circulating high-density lipoproteins in bioengineered human vessels. Elife.

[159] Yoon JK, Kim J, Shah Z, Awasthi A, Mahajan A, Kim YT (2021). Advanced Human BBB-on-a-chip: a new platform for Alzheimer’s Disease Study. Adv Healthc Mater.

[160] Blauwendraat C, Nalls MA, Singleton AB (2020). The genetic architecture of Parkinson’s Disease. Lancet Neurol.

[161] de Rus Jacquet A, Alpaugh M, Denis HL, Tancredi JL, Boutin M, Decaestecker J (2023). The contribution of inflammatory astrocytes to BBB impairments in a brain-chip model of Parkinson’s Disease. Nat Commun.

[162] Balestrino R, Schapira AHV (2020). Parkinson Disease. Eur J Neurol.

[163] Pediaditakis I, Kodella KR, Manatakis DV, Le CY, Hinojosa CD, Tien-Street W (2021). Modeling alpha-synuclein pathology in a human brain-chip to assess blood-brain barrier disruption. Nat Commun.

[164] Lassmann H, Van Horssen J, Mahad D (2012). Progressive multiple sclerosis: pathology and pathogenesis. Nat Rev Neurol.

[165] Nair AL, Groenendijk L, Overdevest R, Fowke TM, Annida R, Mocellin O et al. Human BBB-on-a-chip reveals barrier disruption, endothelial inflammation, and T cell migration under neuroinflammatory conditions. Front Mol Neurosci. 2023;16. 10.3389/fnmol.2023.1250123 PMC10561300 37818458

[166] De Vries HE, Blom-Roosemalen MC, de Boer AG, van Berkel TJ, Breimer DD, Kuiper J (1996). Effect of endotoxin on permeability of bovine cerebral endothelial cell layers in vitro. J Pharmacol Exp Ther.

[167] Mato M, Sakamoto A, Ookawara S, Takeuchi K, Suzuki K (1998). Ultrastructural and immunohistochemical changes of fluorescent granular perithelial cells and the interaction of FGP cells to microglia after lipopolysaccharide administration. Anat Rec.

[168] Zhou H, Andonegui G, Wong CHY, Kubes P (2009). Role of endothelial TLR4 for Neutrophil Recruitment into Central Nervous System microvessels in systemic inflammation. J Immunol.

[169] Lund S, Christensen KV, Hedtjärn M, Mortensen AL, Hagberg H, Falsig J (2006). The dynamics of the LPS triggered inflammatory response of murine microglia under different culture and in vivo conditions. J Neuroimmunol.

[170] Villabona-Rueda A, Erice C, Pardo CA, Stins MF (2019). The Evolving Concept of the blood brain barrier (BBB): from a single static barrier to a heterogeneous and dynamic Relay Center. Front Cell Neurosci.

[171] Macdonald JA, Murugesan N, Pachter JS (2010). Endothelial cell heterogeneity of blood-brain barrier gene expression along the cerebral microvasculature. J Neurosci Res.

[172] Murugesan N, Macdonald JA, Lu Q, Wu SL, Hancock WS, Pachter JS (2011). Analysis of mouse brain microvascular endothelium using laser capture microdissection coupled with proteomics. Methods Mol Biol.

[173] Vanlandewijck M, He L, Mäe MA, Andrae J, Ando K, Del Gaudio F (2018). A molecular atlas of cell types and zonation in the brain vasculature. Nature.

[174] Coomber BL, Stewart PA (1985). Morphometric analysis of CNS microvascular endothelium. Microvasc Res.

[175] Morita S, Furube E, Mannari T, Okuda H, Tatsumi K, Wanaka A (2016). Heterogeneous vascular permeability and alternative diffusion barrier in sensory circumventricular organs of adult mouse brain. Cell Tissue Res.

[176] Cavaglia M, Dombrowski SM, Drazba J, Vasanji A, Bokesch PM, Janigro D (2001). Regional variation in brain capillary density and vascular response to ischemia. Brain Res.

[177] El-Khoury N, Braun A, Hu F, Pandey M, Nedergaard M, Lagamma EF (2006). Astrocyte end-feet in germinal matrix, cerebral cortex, and white matter in developing infants. Pediatr Res.

[178] Nyúl-Tóth Á, Suciu M, Molnár J, Fazakas C, Haskó J, Herman H (2016). Differences in the molecular structure of the blood-brain barrier in the cerebral cortex and white matter: an in silico, in vitro, and ex vivo study. Am J Physiol - Hear Circ Physiol.

[179] Bernier LP, Brunner C, Cottarelli A, Balbi M (2021). Location matters: navigating Regional Heterogeneity of the neurovascular unit. Front Cell Neurosci.

[180] Murugesan N, Demarest TG, Madri JA, Pachter JS (2012). Brain regional angiogenic potential at the neurovascular unit during normal aging. Neurobiol Aging.

[181] Janota CS, Brites D, Lemere CA, Brito MA (2015). Glio-vascular changes during ageing in wild-type and Alzheimer’s disease-like APP/PS1 mice. Brain Res.

[182] Kervezee L, Hartman R, Van Den Berg DJ, Shimizu S, Emoto-Yamamoto Y, Meijer JH (2014). Diurnal variation in P-glycoprotein-mediated transport and cerebrospinal fluid turnover in the brain. AAPS J.

[183] Zhang SL, Lahens NF, Yue Z, Arnold DM, Pakstis PP, Schwarz JE (2021). A circadian clock regulates efflux by the blood-brain barrier in mice and human cells. Nat Commun.

[184] Zhang SL, Yue Z, Arnold DM, Artiushin G, Sehgal A (2018). A circadian clock in the blood-brain barrier regulates Xenobiotic Efflux. Cell.

[185] Schurhoff N, Toborek M (2023). Circadian rhythms in the blood-brain barrier: impact on neurological disorders and stress responses. Mol Brain.

[186] Skapetze L, Owino S, Lo EH, Arai K, Merrow M, Harrington M. Rhythms in barriers and fluids: circadian clock regulation in the aging neurovascular unit. Neurobiol Dis. 2023;181. 10.1016/j.nbd.2023.106120 37044366

[187] Vitaterna MH, King DP, Chang AM, Kernhauser JM, Lowrey PL, McDonald JD (1994). Mutagenesis and mapping of a mouse gene, clock, essential for circadian behavior. Sci (80-).

[188] Hogenesch JB, Chan WK, Jackiw VH, Brown RC, Gu YZ, Pray-Grant M (1997). Characterization of a subset of the basic-helix-loop-helix-PAS superfamily that interacts with components of the dioxin signaling pathway. J Biol Chem.

[189] Ikeda M, Nomura M (1997). CDNA cloning and tissue-specific expression of a novel basic helex-loop-helix/PAS protein (BMAL1) and identification of alternatively spliced variants with alternative translation initiation site. Biochem Biophys Res Commun.

[190] Van Der Horst GTJ, Muijtjens M, Kobayashi K, Takano R, Kanno SI, Takao M (1999). Mammalian Cry1 and Cry2 are essential for maintenance of circadian rhythms. Nature.

[191] Ko CH, Takahashi JS (2006). Molecular components of the mammalian circadian clock. Hum Mol Genet.

[192] Balsalobre A, Damiola F, Schibler U (1998). A serum shock induces circadian gene expression in mammalian tissue culture cells. Cell.

[193] O’Neill JS, Reddy AB (2012). The essential role of cAMP/Ca2 + signalling in mammalian circadian timekeeping. Biochem Soc Trans.

[194] Huang TS, Grodeland G, Sleire L, Wang MY, Kvalheim G, Laerum OD (2009). Induction of circadian rhythm in cultured human mesenchymal stem cells by serum shock and cAMP analogs in vitro. Chronobiol Int.

[195] Ndikung J, Storm D, Violet N, Kramer A, Schönfelder G, Ertych N (2020). Restoring circadian synchrony in vitro facilitates physiological responses to environmental chemicals. Environ Int.

[196] Han K, Mei L, Zhong R, Pang Y, Zhang EE, Huang Y (2020). A microfluidic approach for experimentally modelling the intercellular coupling system of a mammalian circadian clock at single-cell level. Lab Chip.

[197] Brown SA, Zumbrunn G, Fleury-Olela F, Preitner N, Schibler U (2002). Rhythms of mammalian body temperature can sustain peripheral circadian clocks. Curr Biol.

[198] Kaneko H, Kaitsuka T, Tomizawa K (2020). Response to Stimulations Inducing Circadian Rhythm in Human Induced Pluripotent Stem cells. Cells.

[199] Hablitz LM, Plá V, Giannetto M, Vinitsky HS, Stæger FF, Metcalfe T (2020). Circadian control of brain glymphatic and lymphatic fluid flow. Nat Commun.

[200] Del Zoppo GJ (2012). Aging and the neurovascular unit. Ann N Y Acad Sci.

[201] Li Y, Xie L, Huang T, Zhang Y, Zhou J, Qi B (2019). Aging neurovascular unit and potential role of DNA damage and repair in combating vascular and neurodegenerative disorders. Front Neurosci.

[202] Osipova ED, Komleva YK, Morgun AV, Lopatina OL, Panina YA, Olovyannikova RY (2018). Designing in vitro blood-brain barrier models reproducing alterations in Brain Aging. Front Aging Neurosci.

[203] Chen J, Brodsky SV, Goligorsky DM, Hampel DJ, Li H, Gross SS (2002). Glycated collagen I induces premature senescence-like phenotypic changes in endothelial cells. Circ Res.

[204] Yin Y, Zhou Z, Liu W, Chang Q, Sun G, Dai Y (2017). Vascular endothelial cells senescence is associated with NOD-like receptor family pyrin domain-containing 3 (NLRP3) inflammasome activation via reactive oxygen species (ROS)/thioredoxin-interacting protein (TXNIP) pathway. Int J Biochem Cell Biol.

[205] Thambisetty M, Beason-Held LL, An Y, Kraut M, Metter J, Egan J (2013). Impaired glucose tolerance in midlife and longitudinal changes in brain function during aging. Neurobiol Aging.

[206] Mullins RJ, Diehl TC, Chia CW, Kapogiannis D (2017). Insulin resistance as a link between amyloid-Beta and tau pathologies in Alzheimer’s Disease. Front Aging Neurosci.

[207] Fang EF, Lautrup S, Hou Y, Demarest TG, Croteau DL, Mattson MP (2017). NAD + in aging: Molecular mechanisms and translational implications. Trends Mol Med.

[208] James EL, Michalek RD, Pitiyage GN, De Castro AM, Vignola KS, Jones J (2015). Senescent human fibroblasts show increased glycolysis and redox homeostasis with extracellular metabolomes that overlap with those of irreparable DNA damage, aging, and Disease. J Proteome Res.

[209] Karnewar S, Neeli PK, Panuganti D, Kotagiri S, Mallappa S, Jain N (2018). Metformin regulates mitochondrial biogenesis and senescence through AMPK mediated H3K79 methylation: relevance in age-associated vascular dysfunction. Biochim Biophys Acta - Mol Basis Dis.

[210] Jiang T, Cadenas E (2014). Astrocytic metabolic and inflammatory changes as a function of age. Aging Cell.

[211] Ross JM, Öberg J, Brené S, Coppotelli G, Terzioglu M, Pernold K (2010). High brain lactate is a hallmark of aging and caused by a shift in the lactate dehydrogenase A/B ratio. Proc Natl Acad Sci U S A.

[212] Brennand KJ (2013). Inducing cellular aging: enabling neurodegeneration-in-a-dish. Cell Stem Cell.

[213] Miller JD, Ganat YM, Kishinevsky S, Bowman RL, Liu B, Tu EY (2013). Human iPSC-based modeling of late-onset Disease via progerin-induced aging. Cell Stem Cell.

[214] Galatro TF, Holtman IR, Lerario AM, Vainchtein ID, Brouwer N, Sola PR (2017). Transcriptomic analysis of purified human cortical microglia reveals age-associated changes. Nat Neurosci.

